# Ischemia-modified albumin as a possible marker of oxidative stress in patients with telogen effluvium^☆^^☆☆^

**DOI:** 10.1016/j.abd.2020.01.005

**Published:** 2020-05-06

**Authors:** Unsal Savci, Engin Senel, Aynure Oztekin, Mustafa Sungur, Ozcan Erel, Salim Neselioglu

**Affiliations:** a Department of Medical Microbiology, Erol Olcok Education and Research Hospital, Hitit University, Çorum, Turkey; b Department of Dermatology, Faculty of Medicine, Hitit University, Çorum, Turkey; c Department of Urology, Erol Olcok Education and Research Hospital, Hitit University, Çorum, Turkey; d Department of Biochemistry, Ataturk Training and Research Hospital, Ankara, Turkey

**Keywords:** Alopecia, Inflammation, Oxidative stress

## Abstract

**Background:**

Telogen effluvium is the most common form of non-scarring alopecia characterized by diffuse hair loss. Ischemia-modified albumin is a marker of oxidative stress and inflammation.

**Objective:**

The aim of this study was to compare the levels of ischemia-modified albumin of telogen effluvium patients with healthy controls.

**Methods:**

Ninety-one patients diagnosed with telogen effluvium and 35 healthy volunteers were included in the study. Serum ischemia-modified albumin level was determined by a fast-colorimetric method, and albumin cobalt binding test. The results were evaluated statistically.

**Results:**

There was no statistically significant difference between the serum albumin values of patient and control groups (*p* = 0.739). Serum ischemia-modified albumin values were significantly higher in the patients with telogen effluvium than healthy controls (*p* < 0.001).

**Study limitations:**

Body mass index values of the patient and control groups could not be calculated.

**Conclusions:**

To the best of the authors’ knowledge, this is the first clinical study to investigate the role of oxidative stress in the pathogenesis of telogen effluvium using ischemia-modified albumin as a biomarker. Based on the results of the present study, it can be considered that oxidative stress plays an important role in the pathogenesis of telogen effluvium. There is a need for further studies to support the results of this study, to demonstrate the possible effects of oxidative stress, and to investigate the other oxidative stress markers in the pathogenesis of telogen effluvium.

## Introduction

Telogen effluvium (TE) is a type of nonscarring alopecia characterized by often acute and diffuse hair shedding and caused or induced by various acute and chronic diseases and physiological stressful events. The disease is one of the most common causes of diffuse hair loss. Pathogenesis of the disorder is quite heterogenous.^1^ Headington attempted to classify the disease and suggested five different pathogenies: (1) immediate anagen release, (2) immediate telogen release, (3) delayed anagen release, (4) delayed telogen release, and (5) shortened anagen.^2^

Each hair follicle has three cyclic phases: anagen (growth), catagen (regression leading to apoptosis), and telogen (resting). Approximately 100,000 hairs are found on the scalp, of which 10–15% are in the telogen phase and 85–90% in the anagen phase. On average, the anagen phase lasts two to six years, the catagen phase lasts four to six weeks, and telogen phase lasts three to four months in the scalp.^3^ The end of the anagen phase and the onset of the catagen/telogen phase are associated with the biological clock, which is a highly complex phenomenon that arises on a molecular basis in the human body. Various metabolic changes due to pregnancy, malnutrition, and various stressful conditions may affect the biological clock in the hair follicles and abnormally many hair follicles can enter the telogen phase simultaneously. If the growth of a significant part of the hair in the anagen phase stops early due to the effect of any stimulation, it enters the catagen phase, followed by telogen phase, resulting in the formation of TE.^4^ The pathogenesis of TE is unclear and many triggering conditions may play role in the pathogenesis, such as environmental and metabolic factors, including hormones, toxins, cytokines, nutrients, vitamins, and energy deficiencies.^5^

Oxidative stress has been shown to play a key role in these disorders as in many diseases. Presence of oxidative stress has been demonstrated in many dermatological diseases such as seborrheic dermatitis, vitiligo, skin cancers, lichen planus, atopic dermatitis, acne vulgaris, psoriasis, and pemphigus vulgaris.^6^, ^7^, ^8^, ^9^, ^10^, ^11^, ^12^

Various biochemical markers have been detected in oxidative stress and inflammation so far. Ischemia-modified albumin (IMA) is one of these markers. This marker has been used for the detection of myocardial ischemia. IMA levels have been reported to increase in vascular endothelial cell dysfunction and oxidative stress-related diseases. During ischemia, the metal binding capacity of albumin decreases due to free radical damage at the end of the amino terminus (N-terminus) of the metal-binding part.^13^ This new, chemically altered or degraded albumin caused by tissue ischemia is called IMA and is used as a sensitive biochemical marker of ischemia and oxidative stress.^14^

In recent years, high levels of IMA have been indicated to be associated with various diseases based on oxidative stress.^15^ In a limited number of studies investigating IMA levels in dermatological diseases, high levels of IMA have been reported in several dermatological diseases such as vitiligo, psoriasis, and Behçet's.^16^, ^17^, ^18^ Increased median and adjusted IMA levels were detected in alopecia areata patients in the liteature.^19^ In a recent study, it was noted that oxidative stress is closely associated with TE pathogenesis.^20^ In the medical literature there are no studies investigating IMA levels as oxidative stress biomarkers in patients with TE. In the present study, it was hypothesized that the pathogenesis of TE might be associated with IMA.

The aim of this study was to compare IMA levels of TE patients with the healthy control group. To best of the authors’ knowledge, there have been no studies investigating oxidant–antioxidant balance and oxidative stress in the patients with TE in the medical literature. This represents the first clinical study evaluating oxidative stress and IMA levels in the patients with TE.

## Methods

This study included 91 patients (83 females, 8 males) older than 18 years admitted to the dermatology clinic with TE diagnosis and 35 healthy volunteers (31 females, 4 males). The current study was approved by the Ethics Committee of Tokat Gaziosmanpaşa Medicine Faculty under number 19-KAEK-002, carried out in accordance with the Helsinki Declaration. Written informed consent was obtained from all patients and healthy volunteers.

The diagnosis was made by detailed physical examination, patient history (more than 100 hairs lost per day), and positive hair pull test. The hair pull test is strongly positive in TE and is performed by grasping 40–60 scalp hair with thumb and index finger and pulling gently. If more than two to three hairs are removed with traction, the test should be considered as positive.

Other causes of hair loss such as trichotillomania, alopecia areata, cicatricial alopecia, and androgenetic alopecia were ruled out in the patients included and all patients with an additional hair disorder were excluded from the study. The control group comprised volunteers who had no complaints of hair loss and with the same exclusion criteria as the TE patient group.

The exclusion criteria for patient and healthy control groups were as follows: presence of systemic disease, cardiovascular disease, history of surgery, low calorie diet, severe weight loss, active smoking, iron supplementation, presence of menstrual irregularities, pregnancy, lactation, and hair loss caused by drug use. All patients with possible conditions that were likely to alter IMA level were excluded. Venous blood samples were collected after at least 8 h of fasting from the patient and control groups. The samples were centrifuged at 1500 g for 10 min and the serum samples were separated. Separated serum samples were planced in Eppendorf tubes and stored at −80 °C.

The IMA level was measured using the albumin cobalt binding test, a fast-colorimetric method developed by Bar-Or et al.^21^ The method is based on the reduction of the ability of human serum albumin to bind cobalt ions (Co^2+^) depending on ischemia. As a result of the ischemic process, modified albumin was much less bound to Co (II) and the excess (unbound) Co^2+^ amount formed a complex with dithiothreitol, and this complex was measured at 450 nm spectrophotometrically. Plasma albumin level was measured in the autoanalyzer by using the bromocresol green method (Architect Plus C8000; Abbott – United States).

The statistical analysis was performed by using SPSS (SPSS v. 23.0 for Windows – SPSS Inc., Chicago, IL, United States; licensed for Hitit University). Shapiro–Wilk test was used to check the normality assumption for the distribution of the quantitative variables (serum albumin, IMA). For all tested variables, the normality assumption could be considered valid. Student's *t*-test for independent samples and Fisher's exact test were performed to detect possible differences of age and gender between the patient and control groups. A *p*-value < 0.05 was considered statistically significant.

## Results

The mean age was 31.64 ± 12.86 years for the patient group and 32.08 ± 11.60 years for the controls. There was no statistically significant difference between the groups in terms of age and sex (*p* = 0.867 and *p* = 0.736, respectively). There was no statistically significant difference between the two groups in terms of albumin values (*p* = 0.739). The mean IMA value was significantly higher in the patient group (0.77 ± 0.14 g/L) than the control group (0.50 ± 0.09 g/L). There were statistically significant differences between two groups IMA values (*p* < 0.001). Demographic characteristics, albumin, and IMA values and statistical values of the groups are shown in table 1.

**Table 1:** Demographic characteristics of patient and control groups, and serum albumin and IMA levels.

Parameter	Control (*n* = 35)	Patients (*n* = 91)	*p*
Age (mean ± SD, years)	32.08 ± 11.60	31.64 ± 12.86	*p* = 0.867
Gender (male/female)	4/31	8/83	*p* = 0.736
Serum albumin (mean ± SD, g/L)	4.08 ± 0.11	4.08 ± 0.10	*p* = 0.739
IMA (mean ± SD, g/L)	0.50 ± 0.09	0.77 ± 0.14	*p* < 0.001^a^

IMA, ischemia-modified albumin; SD, standard deviation.

a Statistically significant.

## Discussion

TE is a disease characterized by thinning or shedding of the hair in response to the early entry of the hair into the telogen phase. The disorder was first described in 1961 by Kligman. Diagnostic properties of TE are thinning of the affected hair and diffuse hair loss with a strongly positive hair pull test on a scalp that looks normal. A triggering factor causing TE is often found in the patient's history.^1^

IMA was accepted as a marker of myocardial ischemia by the Food and Drug Administration. As a result of exposure to reactive oxygen species in the case of ischemia, the metal (cobalt, copper, zinc) binding capacity of albumin is decreased.^15^ This marker is not only specific for myocardial injury and ischemia. IMA levels have been reported in many diseases such as sepsis, cancer, diabetes, chronic liver disease, and asthma.^22^, ^23^, ^24^, ^25^

IMA has been described as a biomarker of oxidative stress and extensively investigated recently.^21^ The susceptibility, specificity, capacity, and positive and negative predictive values of IMA were detected higher than other studied biomarkers. There have been no studies in the literature investigating oxidative stress in patients with TE. To the best of the authors’ knowledge, a limited number of studies have been conducted evaluating IMA levels in dermatological diseases.

Ataş et al. investigated the importance of oxidative stress in the pathogenesis of vitiligo by measuring the level of IMA; in their study, IMA levels were found to be significantly higher in the patient group than the control group (*p* < 0.0001).^16^

Ozdemir et al. reported that IMA levels were significantly higher in patients with psoriasis compared to healthy controls. They noted that IMA could be produced as an adaptive response to chronic hypoxia and oxidative stress, which is responsible for the systemic inflammation in psoriasis, and that oxidative stress has an importance in the development of psoriasis.^17^ In another study, Ommaet al. evaluated the role of IMA as a biomarker in Behçet's disease activity. In this study, serum IMA levels were significantly higher in Behçet's disease than healthy volunteers (*p* < 0.001).^18^ Like the results of previous dermatological diseases reported in the literature, the present study found that serum IMA levels were statistically higher in TE patients than in healthy controls (*p* < 0.001). Previous studies and the results of the present study may support the relationship between oxidative stress and the pathogenesis of these dermatological diseases.

It is hypothesized that the scalp skin may be chronically exposed to both endogenous and environmental pro-oxidant agents, leading to the formation of reactive oxygen species from damage to cellular components such as nucleic acids, proteins, and cell membrane lipids, and that this may result in deterioration of the antioxidant/oxidant balance. In addition, insufficient antioxidant defense and excessive production of free radicals may contribute to the formation of oxidative stress.^26^ It is important to emphasize that there is an increase in IMA levels in cases of hypoxia, acidosis, and tissue damage caused by free radicals.^17^ IMA levels may be higher in TE patients as increased oxygen radicals affect the structure of albumin. The limitation of the present study was that body mass index values of patient and control groups could not be calculated.

## Conclusions

Based on the results of this study, oxidative stress may play an important role in the pathogenesis of TE. This is the first clinical study to investigate the role of oxidative stress in the pathogenesis of TE using IMA as a biomarker. In addition, this study may provide significant evidence that antioxidant therapy would be useful in TE. Further studies are needed to support the results of this study, to demonstrate the probable effects of oxidative stress, and to investigate the other oxidative stress markers in the pathogenesis of TE.

## Financial support

None declared.

## Authors’ contributions

Unsal Savci: Statistical analysis; approval of the final version of the manuscript; conception and planning of the study; drafting and editing of the manuscript; collection, analysis, and interpretation of data; intellectual participation in the propaedeutic and/or therapeutic conduct of the studied cases; critical review of the literature.

Engin Senel: Conception and planning of the study; collection, analysis, and interpretation of data; critical review of the literature; critical review of the manuscript.

Aynure Oztekin: Conception and planning of the study; collection, analysis, and interpretation of data; participation in the study design; critical review of the literature.

Mustafa Sungur: Statistical analysis; drafting and editing of the manuscript.

Ozcan Erel: Conception and planning of the study; drafting and editing of the manuscript; participation in the study design.

Salim Neselioglu: Conception and planning of the study; participation in the study design; critical review of the literature.

## Conflicts of interest

None declared.
